# An Undetected Pheochromocytoma Leading to Fulminant Adrenergic Myocarditis Complicated by Cardiogenic Shock

**DOI:** 10.1210/jcemcr/luad142

**Published:** 2023-12-01

**Authors:** Nam Q Tran, Hieu T N Tran, Thang V Tran, Thuan T Nguyen

**Affiliations:** Department of Endocrinology, University of Medicine and Pharmacy at Ho Chi Minh City, Ho Chi Minh City, 700000, Viet Nam; Department of Endocrinology, University Medical Center at Ho Chi Minh City, Ho Chi Minh City, 700000, Viet Nam; Department of Endocrinology, University Medical Center at Ho Chi Minh City, Ho Chi Minh City, 700000, Viet Nam; Department of Endocrinology, University of Medicine and Pharmacy at Ho Chi Minh City, Ho Chi Minh City, 700000, Viet Nam; Department of Endocrinology, University Medical Center at Ho Chi Minh City, Ho Chi Minh City, 700000, Viet Nam; Department of Endocrinology, University Medical Center at Ho Chi Minh City, Ho Chi Minh City, 700000, Viet Nam

**Keywords:** pheochromocytoma, pheochromocytoma crisis, fulminant adrenergic myocarditis, Brugada syndrome

## Abstract

Pheochromocytomas are rare catecholamine-secreting neuroendocrine tumors. Their episodic nature is correlated with abrupt catecholamine release and clinical manifestations that mimic other vascular conditions, leading to delayed diagnosis and potentially life-threatening complications, such as acute myocarditis and pheochromocytoma crises. In this report, we described the case of fulminant adrenergic myocarditis-induced cardiogenic shock requiring extracorporeal membrane oxygenation support in a Vietnamese middle-aged man with a 5-year history of Brugada syndrome, hypertension, and previously undiagnosed pheochromocytoma. After stabilization, the patient was medically treated with a combination of α- and β-blockers before undergoing laparoscopic right adrenalectomy.

## Introduction

Pheochromocytomas are catecholamine-secreting tumors that originate from chromaffin cells of the autonomic nervous system. These conditions can prove fatal if undetected or mismanaged. The secreted catecholamines explain the clinical manifestations of these tumors, such as hypertension, headache, palpitations, sweating, and dyspnea during hypertensive episodes. Due to the unpredictable release of catecholamines, pheochromocytoma presentation can range from clinically asymptomatic to life-threatening crises such as pheochromocytoma crisis (PCC) [1].

PCC is characterized by the rapid and severe presentation of catecholamine-induced hemodynamic instability, resulting in end-organ dysfunction [1]. This is an uncommon and highly fatal complication of pheochromocytomas. The overall rate of mortality from PCC is approximately 13% to 15%, with the heart being the most commonly damaged organ in 99% of PCC cases [1, 2]. Fulminant myocarditis is a myocardial inflammatory condition depicted by sudden onset and rapid progression, leading to serious outcomes, including death. This rare manifestation of pheochromocytoma was first reported in 1990 by Sardesai et al and was regarded as adrenergic or catecholamine-induced myocarditis [3]. Autopsy studies have indicated that more than 50% of patients with pheochromocytoma exhibit active myocarditis with left ventricular (LV) dysfunction and pulmonary edema, as indicated by structural alterations resembling acute myocarditis [4].

In this report, we described the case of a middle-aged patient with undetected pheochromocytoma-induced fulminant adrenergic myocarditis complicated by cardiogenic shock.

## Case Presentation

**Figure 1.**
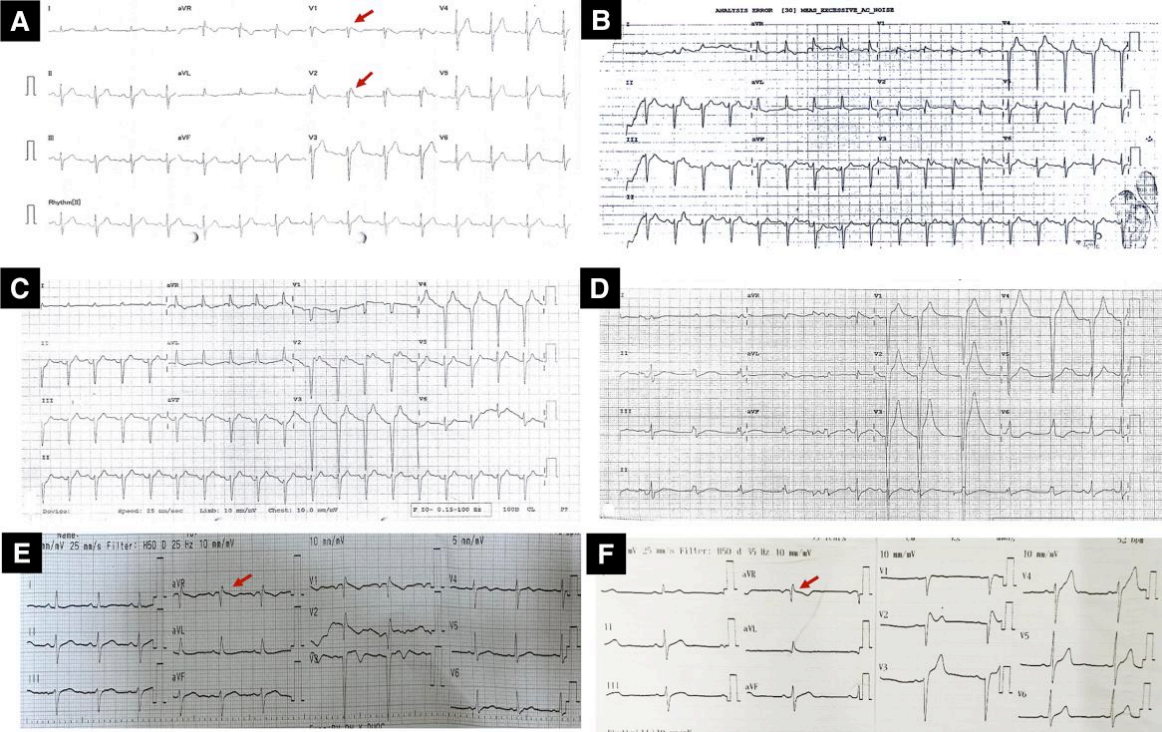
ECG changes of the patient. A, Previous ECG (in 2020) showing Brugada syndrome type I pattern: coved ST-segment elevation in V1 and V2. B, ECG at presentation: sinus tachycardia, Q waves in V1-V3. C, ECG after a coronary angiogram showing left axial deviation, ST-elevation in multiple leads, Q waves in V1-V4. D. ECG after ECMO cessation. (E) and (F). ECG at discharge and 1-month follow-up showing Brugada pattern at V1.

A 54-year-old man presented to a rural hospital with acute onset of chest tightness, palpitations, dyspnea, and diaphoresis. On physical examination, his systolic blood pressure was 240 mmHg, and heart rate was 110 beats/min. The echocardiogram (ECG) showed sinus tachycardia and Q waves in V1-V4 (Fig. 1B). Blood test results confirmed significant elevations of troponin I level from 48.4 to 20 259 µg/L and N-terminal pro B-type natriuretic peptide (NT-pro BNP) level of 13 816 ng/L (Table 1). The patient, with initially suspected myocardial infarction, was transferred to an urgent cardiac catheterization, which showed no coronary artery stenosis.

**Table 1.** Blood tests at presentation

Parameter	Reference range	Result
Troponin I	<17.5 pg/mL (<17.5 μg/L)	48.8 → 20 259 pg/mL (48.8 → 20 259 μg/L)
NT-pro BNP	<125 pg/mL (<125 ng/L)	13 816 pg/mL (13 816 ng/L)
WCC	4.5-10 × 10^9^/L	17.3 × 10^9^/L
* Neutrophils*	*1.4-6.5* × *10^9^/L*	*14.61* × *10^9^/L*
* Lymphocytes*	*1.2-3.4* × *10^9^/L*	*1.44* × *10^9^/L*
* Eosinophils*	*0-.7* × *10^9^/L*	*.09* × *10^9^/L*
* Monocytes*	*.1-.6* × *10^9^/L*	*1.13* × *10^9^/L*
C-reactive protein	<5.0 mg/L (<47 nmol/L)	36 mg/L (342 nmol/L)
Pro-calcitonin	<.5 ng/mL <.5 *μ*g/L)	8.1 ng/mL (8.1 *μ*g/L)
Creatinine	.7-1.5 ng/mL (53.38-114.38 μmol/L)	1.84 ng/mL (140.30 μmol/L)
eGFR	>60 mL/min/1.73 m^2^	51 mL/min/1.73 m^2^
Glucose	3.9-6.4 mmol/L	18.51 mmol/L

Abbreviations: eGFR, estimated glomerular filtration rate; NT-pro BNP, N-terminal pro B-type natriuretic peptide; WCC, white cell count.

Following cardiac catheterization, the patient became hemodynamically unstable with fever 39.9 °C, tachycardia (heart rate: 140 beats/min), hypotension (blood pressure: 70-90/50 mmHg) requiring 3 high-dose vasopressors (epinephrine, norepinephrine, and dobutamine), and tachypnea (respiratory rate: 40 breaths/min). Repeated ECG showed ST-elevation in V1-V4 (Fig. 1C). Bedside echocardiography revealed diffuse hypokinesis of the LV wall with an estimated LV ejection fraction (LVEF) of 25%. The chest x-ray revealed no cardiopulmonary changes. The patient was diagnosed with cardiogenic shock secondary to suspected acute myocarditis. Viral studies, including for COVID-19, were negative. Endomyocardial biopsy (EMB) and cardiac magnetic resonance imaging (MRI) were not performed at this point owing to rapid deterioration. Extracorporeal membrane oxygenation (ECMO) was urgently initiated. The patient gradually stabilized under ECMO support, and vasopressors were progressively discontinued, with no hypertensive episodes. Repeated echocardiogram revealed improved LVEF, from 25% to 38%. The patient was extubated after 3 days and ECMO was weaned off after 4 days. However, after ECMO discontinuation, his blood pressure suddenly increased again.

## Diagnostic Assessment

Due to wide blood pressure fluctuations, a pheochromocytoma was considered. His medical history was re-reviewed. Five years prior to this presentation, the patient had reported chest tightness, palpitation, and diaphoresis persisting for 15 minutes while drinking traditional tea. Consequently, an ECG was performed, showing a Brugada type 1 pattern (Fig. 1A). He was diagnosed with Brugada syndrome and has worn an implantable cardioverter defibrillator (ICD) since. After a year, he was diagnosed with hypertension and was treated with losartan, amlodipine, and metoprolol. His blood pressure was extremely labile, and he was prescribed antihypertensive medications intermittently over the past 4 years. The patient had no other significant family or social history.

**Figure 2.**
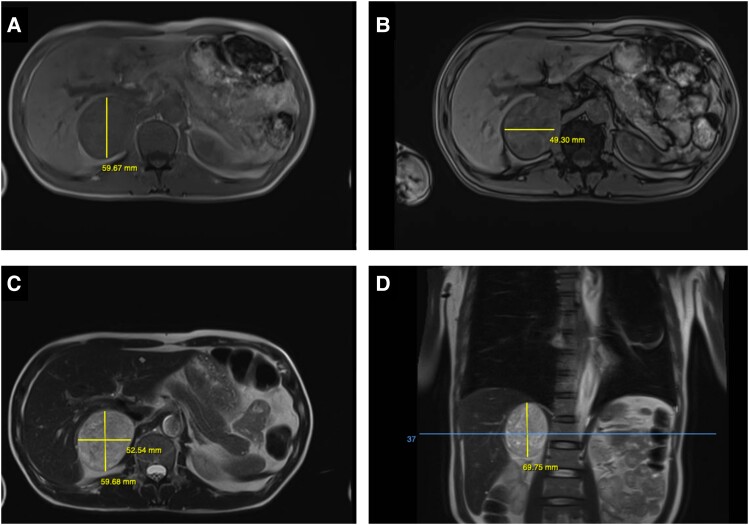
Abdominal MRI images demonstrating a well-defined right adrenal tumor with a heterogenous soft tissue density shadow. A and B, Axial T1-weighted images showed a mass measured 6 × 5 cm with low signal intensity. C, Axial T2-weighted image and D, coronal T2-weighted image showed increased signal intensity.

**Figure 3.**
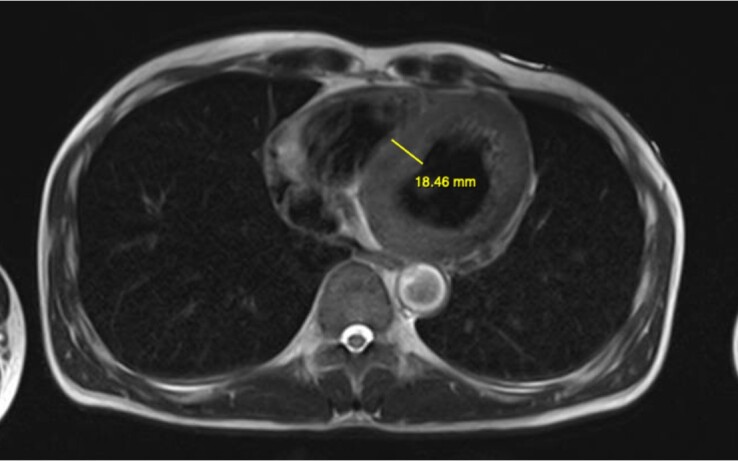
Cardiac MRI showing myocardial edema with the left ventricular wall thickening (8-18 mm) in axial T2-weighted image.

With this consideration of pheochromocytoma, an urgent MRI of the abdomen was ordered, while awaiting fasting plasma metanephrines. The MRI showed a 6 × 5 × 7 cm right heterogenous adrenal mass (Fig. 2). Concurrently, a cardiac MRI was also performed, which revealed myocardial edema of the LV, hypokinesis of the apex with an LVEF of 42%, a global signal intensity ratio (myocardium/skeletal muscles) of 2.6, and moderate pericardial effusion (Fig. 3). These findings supported the diagnosis of catecholamine-induced fulminant myocarditis leading to cardiogenic shock. Blood tests indicated increased levels of catecholamine: metanephrine level, 1696 pg/mL (8921 pmol/L) (reference range, < 90 pg/mL, < 473 pmol/L); normetanephrine level, 3790 pg/mL (20 693 pmol/L) (reference range, < 196 pg/mL, < 1070 pmol/L) (Table 2). Other biochemical tests excluded primary aldosteronism and adrenal Cushing syndrome.

**Table 2.** Biochemical results confirming the diagnosis of pheochromocytoma

Parameter	Reference range	Result	
		Pre-adrenalectomy	Post-adrenalectomy
Metanephrine	<90 pg/mL (<473 pmol/L)	1696 pg/mL (8921 pmol/L)	65 pg/mL (341 pmol/L)
Normetanephrine	<196 pg/mL (<1070 pmol/L)	3790 pg/mL (20 693 pmol/L)	116 pg/mL (633 pmol/L)
Adrenaline	<100 pg/mL (<545 pmol/L)	983 pg/mL (5366 pmol/L)	
Noradrenaline	<600 pg/mL (<3546 pmol/L)	1352 pg/mL (7991 pmol/L)	
Dopamine	<100 pg/mL (<652 pmol/L)	401 pg/mL (2617 pmol/L)	

## Treatment

**Figure 4.**
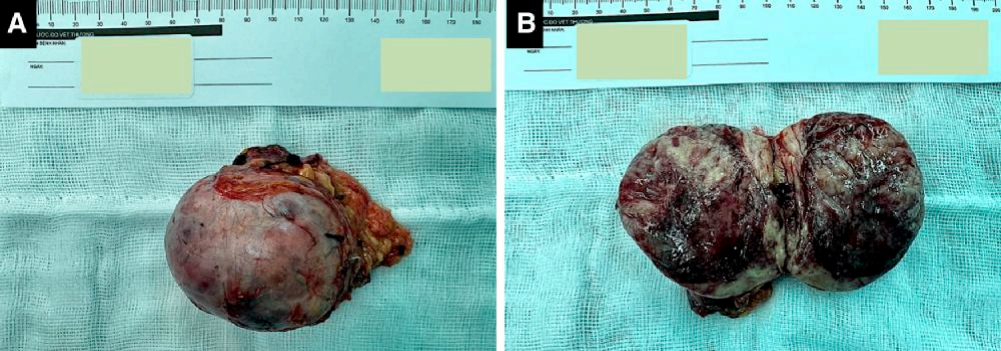
A, Gross anatomy of the right solid extracted tumor measured 6 × 5 × 7 cm. B, A cross-sectional of the tumor with areas of hemorrhage and necrosis.

The patient was treated with α-adrenergic blocker, doxazosin, at a starting dose of 4 mg, titrated up to 8 mg daily. As the patient experienced postural hypotension and valsartan 40 mg daily was also simultaneously administered for the management of his heart failure, the doxazosin dose was reduced to 6 mg daily until the operation. Subsequently, metoprolol 25 mg daily was added to control his heart rate. Repeated preoperative echocardiography revealed significant improvement of LVEF (from 42% to 68%). Ultimately, he underwent a successful right laparoscopic adrenalectomy (Fig. 4).

## Outcome and Follow-Up

**Figure 5.**
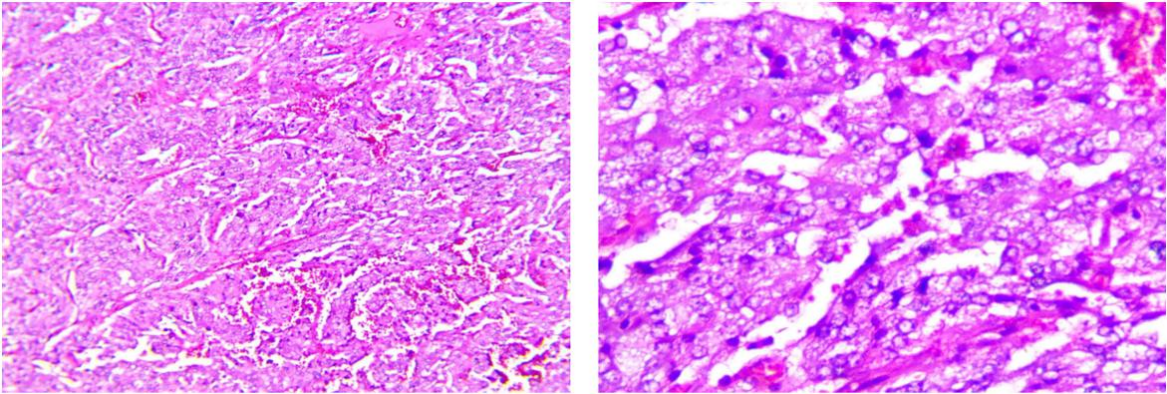
Microscopic features of the right pheochromocytoma showing a Zellballen pattern, with a PASS score of 0.

The patient recovered well and was discharged without antihypertensive medications. At the 1-month follow-up visit, his symptoms had resolved, and his blood pressure was normalized. The metanephrine and normetanephrine were normal (Table 2). Pathological studies confirmed the diagnosis of pheochromocytoma with a Pheochromocytoma of the Adrenal gland Scaled Score (PASS score) of 0 (Fig. 5). Genetic screenings for *SDHB, SDHD, VHL,* and *RET* mutations were negative.

## Discussion

The case demonstrated an unusual presentation of pheochromocytoma as fulminant myocarditis-induced PCC in a patient with a 5-year history of Brugada syndrome and hypertension that required ECMO for stabilization and survival. First, the case reinforced the salience of early recognition of pheochromocytoma. Despite the symptoms suggestive of pheochromocytoma and the paroxysmal attacks in the past 5 years, no investigations were performed to screen for potential pheochromocytoma. He was diagnosed with hypertension and managed with 3 antihypertensive medications, including β-blockers. His blood pressure was too labile, and his antihypertensive medications were prescribed intermittently. Altogether, this resulted in a 5-year delay in diagnosis. Clinicians should pay heed to potential pheochromocytoma in patients presenting with headaches, palpitations, chest pain, diaphoresis, and hypertension. Their presence in patients with hypertension, especially those with oscillating blood pressure, should arouse immediate suspicion of pheochromocytoma.

PCC can be classified into type A and B [1]. Type A crisis includes PCC cases without sustained hypotension. Contrarily, type B crisis, also known as pheochromocytoma multisystem crisis (PMC), is a more severe presentation with sustained hypotension or shock. In a systematic review assessing PCC outcomes, type B crises had a higher mortality rate (29.7%) than type A (9.9%) [2]. The patient in our report had a type B crisis without a known history of pheochromocytoma and a higher mortality risk. The catecholamine profile of adrenal tumors provides a clinical picture of pheochromocytomas. Patients with epinephrine- or dopamine-secreting tumors are likely to present with hypotension or cardiogenic shock since β-adrenergic stimulation overrides α-adrenergic stimulation [4]. Therefore, their risk of PMC or Type B crisis is higher. This is consistent with our patient, who exhibited high catecholamine levels, including epinephrine and dopamine.

Autopsy studies have estimated that more than 50% of patients with pheochromocytoma die of focal myocardial necrosis and inflammation, mainly contributing to LV dysfunction [4, 5]. Increased circulating catecholamines and their oxidation products may damage myocardial cells, leading to focal degeneration, contraction band necrosis, inflammation, interstitial fibrosis, and downregulation of adrenoceptors, causing suboptimal myofiber function and fewer contracting units [5]. Most case reports present patients with adrenergic myocarditis experiencing with fever on admission, which may result from cytokine production, such as interleukin-6 from pheochromocytoma cells [5]. EMB is usually indicated to diagnose myocarditis by identifying infiltrating cell types. Patients with adrenergic myocarditis may exhibit histopathological features of multifocal contraction and necrosis with patchy infiltration of inflammatory cells like lymphocytes and neutrophils [3, 6]. However, obtaining samples in cases of fulminant myocarditis is challenging. In some instances, myocardial inflammation can be patchy, rendering EMB results inconclusive when tissue specimens are collected from unaffected areas [7]. Clinically suspected myocarditis can be considered based on clinical presentation and diagnostic criteria, including changes in ECG (AV blocks, bundle branch blocks, ST/T wave changes, arrhythmia, abnormal Q waves), increased cardiac biomarkers, functional and structural abnormalities on cardiac MRI, together with fever, acute onset of chest pain, dyspnea, cardiac failure signs, palpitation, and unexplained cardiogenic shock [8]. More identified features parallel higher clinical suspicion. Due to the patient's unstable condition, we did not perform EMB. Still, the diagnosis of fulminant myocarditis could be made based on the acute onset of presenting symptoms, significantly elevated troponin and NT-pro BNP levels, ST changes on the ECG, and unexplained abnormality of LV dysfunction leading to cardiogenic shock. Additionally, the diagnosis was supported by a cardiac MRI showing myocardial edema. Although fulminant adrenergic myocarditis has been reported, this remains an extremely rare yet life-threatening manifestation of pheochromocytoma. Fulminant adrenergic myocarditis-induced PCC cases are even lower; and none involve patients with Brugada syndrome.

The management of fulminant adrenergic myocarditis entails supportive therapy, preoperative medical management, and tumor removal. Mechanical support with ECMO has been gradually recognized as a supportive therapy for patients with acute cardiomyopathy-induced PCC. Under life-threatening circumstances, ECMO can stabilize a patient's hemodynamics and allow sufficient time for subsequent investigations and treatment. Zhou et al reviewed 23 reports on ECMO for the management of catecholamine-induced cardiogenic shock, which showed that 16 patients achieved stable vital signs when ECMO was provided promptly, enabling subsequent tumor resection [9]. This article aims to highlight the effectiveness of ECMO in catecholamine-induced life-threatening conditions. Rapid recovery of cardiac function can be achieved if prompt treatment is provided during the attack.

In addition, catecholamine-induced PCC may be severe in young Caucasian female individuals but perhaps more so in middle-aged Asian male individuals. For instance, Chao et al reported 4 Asian patients with PCC who required the application of ECMO, 3 of whom were middle-aged male patients [9]. This may be explained by broad Sino-European differences in the genetic landscape and the clinical presentation of pheochromocytoma and paraganglioma [10]. This is the very first case report of a Vietnamese patient with a PCC in the literature, and we hope to supplement this information with future studies. In the future, we aim to conduct a multicenter study in this regard for Asian patients with pheochromocytoma.

## Learning Points

- Clinicians should be alert to the suspicion of pheochromocytoma when evaluating patients with nonischemic, nonvalvular cardiomyopathy, labile blood pressure, or cardiogenic shock of unknown etiology.
- Pheochromocytomas should be recognized as one of the causes of acute or fulminant myocarditis.
- Fulminant adrenergic myocarditis is an extremely rare but life-threatening condition that potentially leads to a PCC, an endocrine emergency that requires urgent management.
- Timely mechanical support with ECMO can be effective in PCC if provided promptly.

## Data Availability

Original data generated and analyzed during this study are included in this published article.

## Abbreviations

ECG: electrocardiography
ECMO: extracorporeal membrane oxygenation
EMB: endomyocardial biopsy
LV: left ventricular
LVEF: left ventricular ejection fraction
MRI: magnetic resonance imaging
NT-pro BNP: N-terminal pro B-type natriuretic peptide
PASS: Pheochromocytoma of the Adrenal gland Scaled Score
PCC: pheochromocytoma crisis
PMC: pheochromocytoma multisystem crisis
